# The Helping Hand

**Published:** 1873-12

**Authors:** 


### "THE HELPING HAND"

is an institution at 316 Water street, New York, for the reclamation and encouragement of men who have fallen into temptation, and, being in destitute circumstances, are in immediate need of help. Let those who have a pitying heart try and send them some help in the way of old clothing, food or money.

OLD NEWSPAPERS, Magazines and Pictorials. You have many of them about the house. You have looked them over once and thought you would do it again, but you never will. There is a use for them which is worth more than money. Gather them all up and send the picture papers to lunatic asylums, and the others to poverty-stricken homes, to hospitals and prisons, where they will while away many an hour of sadness in the long, cold winter nights. Many a load of sorrow would be lightened thereby.

During the last official year 5,000 night lodgings have been given, over ten thousand meals afforded, besides fuel, and lights, and rents, for the sum of two thousand dollars—equal to giving a bed to sleep on to the houseless ones, or a dinner to those who have nothing to eat, to fifteen thousand persons. Thus, a single dollar provides a night's lodging or a comfortable dinner to seven poor creatures, who haven't a mouthful to eat or a pillow or pallet to rest upon, and who are to that extent snatched from temptation, and sin, and crime. This strikingly shows how much good a little money does when judiciously employed by such grand men as George J. Mingens—a man who has these many years consecrated himself wholly to the poor of New York, going into their garrets, and cellars, and dens, and almost living with them, that he may personally know what they need, how much they need, and the very least amount of money is required to meet each individual want.

Many a dollar is given at the doors of New Yorkers to persons asking for the means to procure a night's lodging or a single meal, but if sent to Rev. George J. Mingens, 316 Water street, it would do seven times as much good, and always to the deserving, but almost never is it given to such at the street doors. Let the truly benevolent think of this.

